# How a broken vertebra can lead to a fatal hemorrhage: a case report

**DOI:** 10.1186/s12245-024-00594-5

**Published:** 2024-02-23

**Authors:** Roxanne A. W. Ploumen, Martin R. van Wezenbeek, Paul C. P. H. Willems, Suzanne C. Gerretsen, Jan A. Ten Bosch

**Affiliations:** 1 Department of Trauma Surgery, Maastricht University Medical Centre+, Maastricht, The Netherlands; 2 Department of Surgery, Maastricht University Medical Centre+, Maastricht, The Netherlands; 3 Department of Trauma Surgery, Zuyderland Medical Center, Heerlen, The Netherlands; 4 Department of Orthopedics, Maastricht University Medical Centre+, Maastricht, The Netherlands; 5 CAPHRI – Care and Public Health Research Institute, Maastricht University, Maastricht, The Netherlands; 6 Department of Radiology, Maastricht University Medical Centre+, Maastricht, The Netherlands

**Keywords:** Elderly, Spinal fracture, Hemorrhage, Diffuse idiopathic skeletal hyperostosis, Emergency medicine

## Abstract

**Background:**

Unintentional falls are common among the elderly and given the expected increase of the aging population, these falls contribute to a high number of admissions to the emergency department. Relatively low-energy trauma mechanisms can lead to serious injuries in the elderly, with contributing factors being comorbidities, medication use and degenerative abnormalities.

**Case presentation:**

A 94-year-old female suffered an unintentional fall at home. Upon arrival of the ambulance at her house she was hemodynamically stable and mobilized to the gurney with assistance. During primary survey at the emergency department, her blood pressure and oxygen saturation decreased, she was not able to move her legs anymore and lost consciousness. A full-body CTA was performed, which showed a fracture through the vertebral body of L2 with significant dislocation and a large active bleeding of the corpus, extending to the retroperitoneum and the epidural space. Despite resuscitation, her vital signs deteriorated and given the severe abnormalities on CTA, it was decided to discontinue further treatment, after which she deceased. The performed CTA and an x-ray from 2016 suggested diffuse idiopathic skeletal hyperostosis, which might have contributed to the severity and instability of the vertebral fracture. Mobilization after the fall might have increased the dislocation of the fracture. The use of oral anticoagulants worsened the subsequent bleeding and the extension to the epidural space caused the paralysis of the legs.

**Conclusions:**

It is important to be aware of the possible serious consequences of unintentional falls in the elderly population and to provide strict immobilization of the spinal column until proper imaging.

## Background

Unintentional falls are common in the elderly population, with an incidence of 33% of the people aged 65 years or older, representing 105,000 elderly visiting the emergency department each year in the Netherlands [1]. Subsequently, falls are the leading cause of injury-related death in the elderly and contribute to high numbers of hospital admissions [2]. Moreover, given the expected increase of elderly in the general population, the health care-related costs continue to expand [3]. Previous studies emphasize how relatively low-energy trauma mechanisms in the elderly can lead to severe injuries. Contributing factors to the severity of the injuries are degenerative abnormalities, comorbidities, and medication use [4, 5]. Vertebrae, wrists, and hips are most frequently affected. Vertebral fragility fractures occur more often in women, and are associated with an increased level of morbidity and mortality [6, 7]. The majority of these fractures are located between the tenth thoracic and fourth lumbar vertebra [8]. In addition to degenerative conditions that increase with age, such as osteoporosis, other conditions can cause the spine to be more prone to fracture, such as ankylosing spondylitis or diffuse idiopathic skeletal hyperostosis [9].

We report the case of a fall in a 94-year-old in which the severity of initial injury was underestimated compared to the eventual outcome in the emergency department.

### Case presentation

A 94-year-old female was presented at the emergency department after an unwitnessed fall at home. She still lived on her own and mobilized inside her apartment with help of a walking aid. Her medical history reports congestive heart failure, atrial fibrillation, and hypercholesterolemia, for which she used a beta-blocker, an ACE-inhibitor, a coumarin derivative, and a statin. Furthermore, in 2011, she had been diagnosed with hyperuricemia and gout, for which she received allopurinol.

**Fig. 1:**
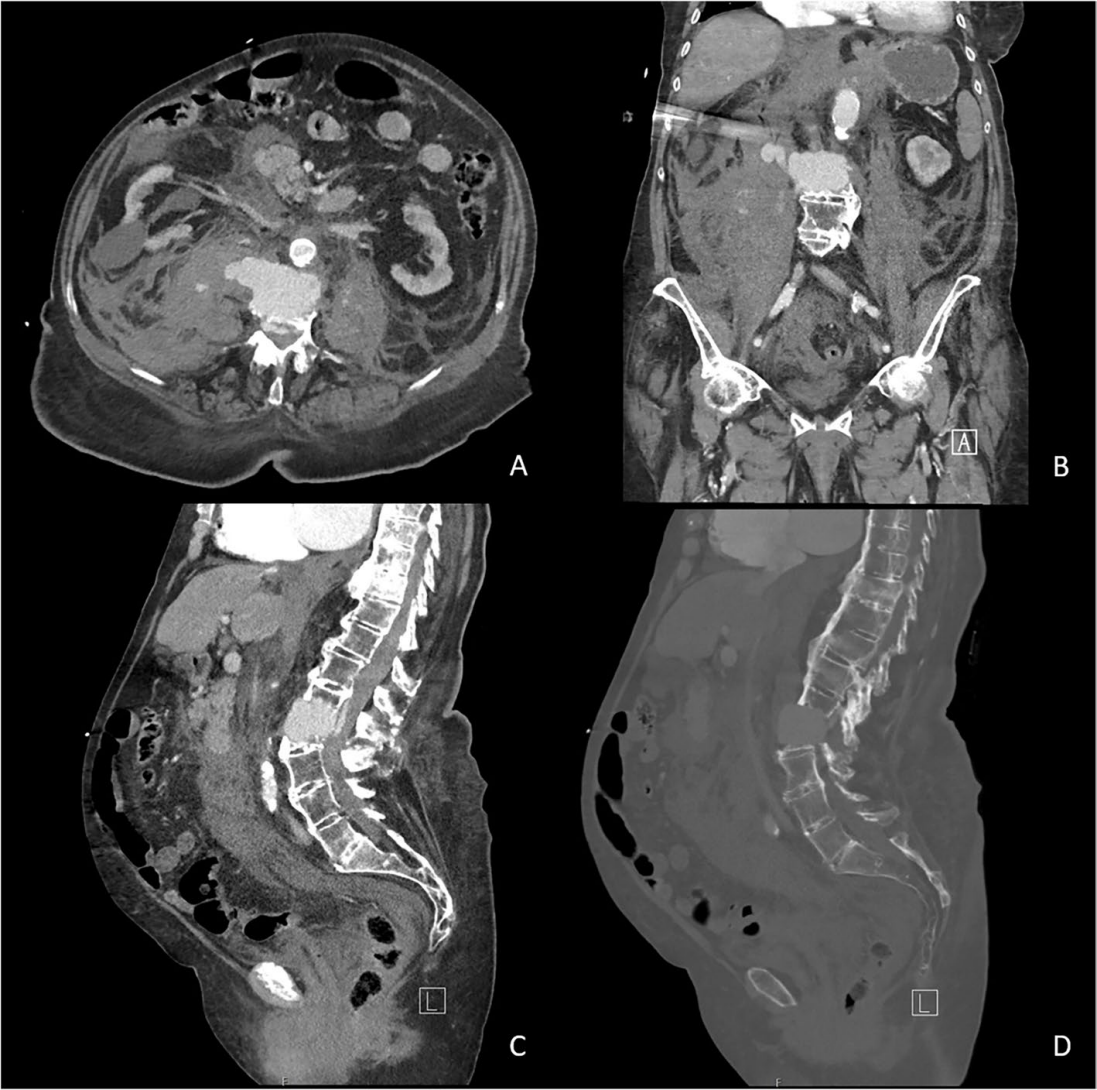
Transverse (**A**), coronal (**B**), and sagittal (**C**, **D**) plane CT slices of the vertebral bleeding. **A**, **B** Active bleeding toward the retroperitoneum with a larger hematoma on the right side, but active contrast extravasation on both sides. **C** Active bleeding extending to the epidural space. **D** CT slice with bone window setting showing substantial dislocation of the fracture and extensive calcification of the anterior ligament of the spine

**Fig. 2:**
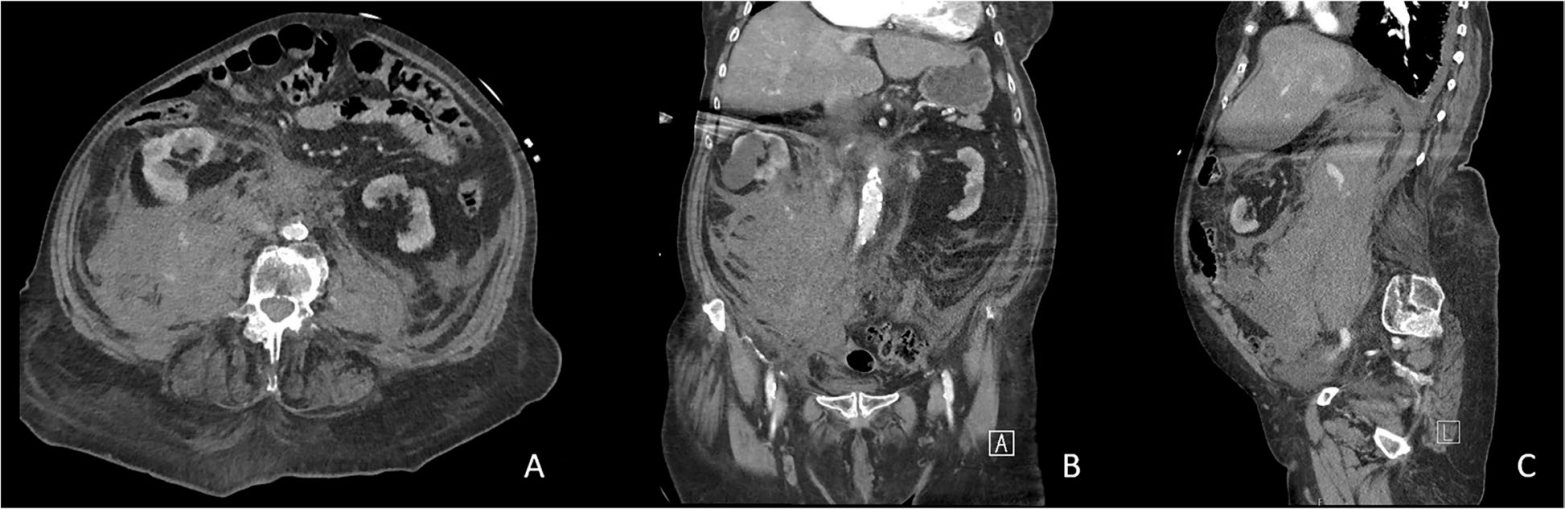
Transverse (**A**), coronal (**B**), and sagittal (**C**) plane CT slices of the extension of the vertebral bleeding into the retroperitoneum with mass effect causing a ventral displacement of the right kidney

The ambulance reported that at the time of their arrival, the patient was conscious. She had fallen when she was alone at home, and her family was able to reach her by phone after one hour. There was no (witnessed) loss of consciousness; however, she suffered from retrograde amnesia and there was suspected head injury. Due to the initial estimation of a low-energy fall, the patient walked to the gurney with assistance of the ambulance nurses; however, after she started complaining of back pain, it was decided to immobilize the spine by transporting her in a vacuum mattress. During transport to the hospital, blood pressure decreased down to 90/50 mmHg, which initially responded to a fluid challenge with 500 ml of Ringer’s lactate. At the emergency department, she underwent a trauma survey according to the principles of the Advanced Trauma and Life Support. Upon arrival, her blood pressure was normal (112/59 mmHg) with tachycardia (137/min), tachypnea (30/min), and she was fully conscious (GCS 15). Within a few minutes, oxygen saturation and blood pressure significantly decreased to 90% and 82/41 mmHg respectively, whereupon a non-rebreathing mask with 15 L oxygen was applied and fluid resuscitation was started using 2 L Ringer’s lactate under pressure. Blood pressure remained low and her consciousness decreased. Plain radiographs of the chest and pelvis showed no abnormalities that could explain a hypovolemic shock. Laboratory findings showed a decreased hemoglobin of 4.4 mmol/L with an INR of 6.0. Prothrombin Complex Concentrate, vitamin K, and tranexamic acid were administered to attenuate blood loss and two units of packed red blood cells were given. Focused Assessment with Sonography in Trauma (FAST) did not show intra-abdominal free fluid. Neurological examination showed a complete paralysis of the lower extremities. A full-body computed tomography angiography (CTA) scan was performed, which showed a completely unstable Chance-type fracture through both the vertebral body and posterior complex of L2, with significant anterior distraction resulting in hyperlordosis. In addition, a large active bleeding was seen, extending to the retroperitoneum and the epidural space (Fig. 1). The extent of the retroperitoneal hematoma caused a mass effect with ventral dislocation of the right kidney (Fig. 2). In addition, a significant calcification of the anterior ligament of the spine was observed (Fig. 1). No intracerebral or intrathoracic post-traumatic abnormalities were detected.

**Fig. 3:**
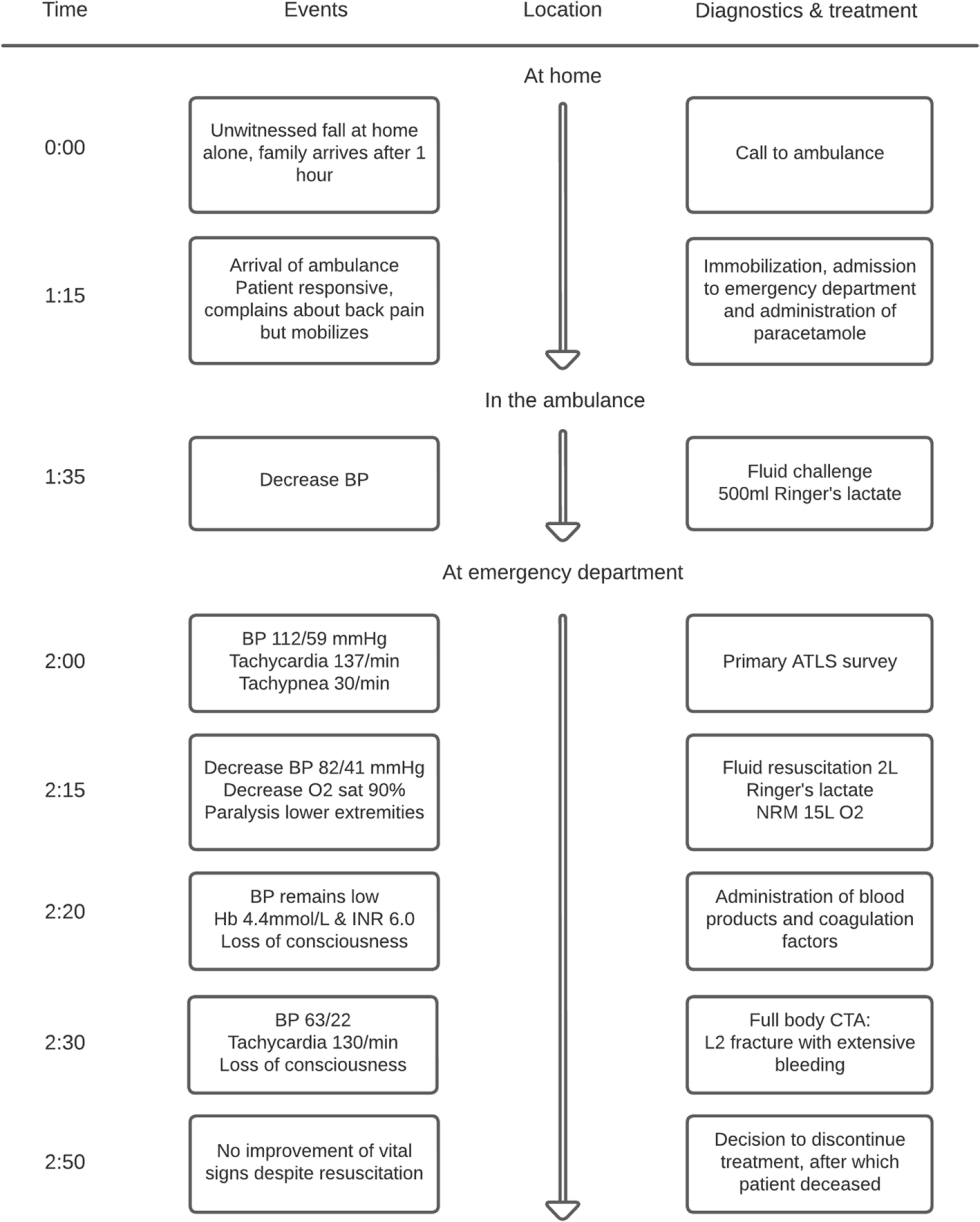
Global timeline of events, diagnostics and treatment. *BP* blood pressure, *ATLS* advanced trauma life support, *O*_*2*_ oxygen, *NRM* non-rebreathing mask, *Hb *hemoglobin*, INR* international normalized ratio, *CTA* computed tomography angiography

After CT imaging, her blood pressure kept decreasing despite resuscitation with in total 3 L of Ringer’s lactate and two units of packed red blood cells. The family was called, and it was decided to make a do-not-resuscitate decision. Given the age, prolonged duration of hypovolemia and subsequent hypoperfusion of the brain, in combination with the neurological and CTA findings, further treatment was discontinued. The patient deceased several minutes after discontinuation of treatment. Figure 3 presents a global timeline of events.

**Fig. 4:**
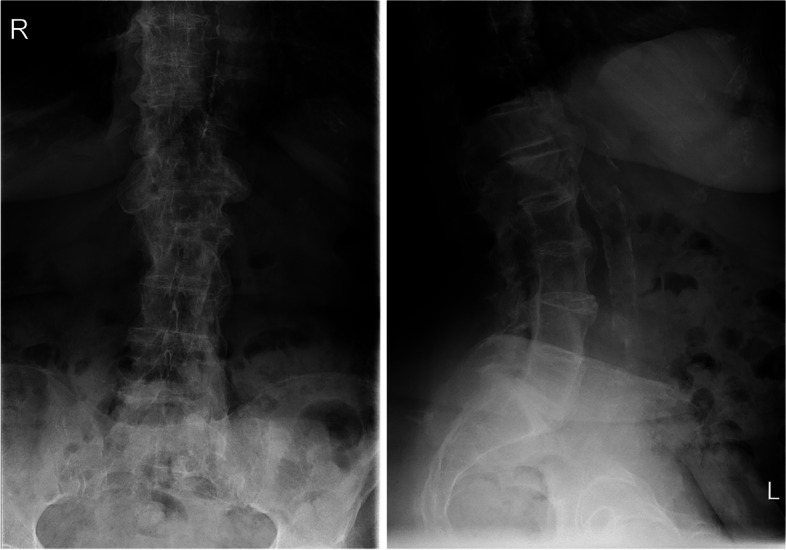
Lumbar spine X-ray in 2016, showing severe degenerative changes and calcification of the anterior ligament, suggesting DISH

The exact origin of the bleeding is difficult to pinpoint, and this degree of bleeding is rare in case of a vertebral fracture. Based on the CT images, no further injury to the abdominal aorta, inferior vena cava, or the lumbar arteries was observed. The location of the bleeding most likely concerned the cancellous bone of the vertebrae, and the severity most probably has been increased by the high INR due to anticoagulant use. In addition, signs of extensive calcifications of the anterior ligament suspected of diffuse idiopathic skeletal hyperostosis were seen on both CT and previous conventional lumbar spine imaging in 2016 (Fig. 4).

## Discussion

The number of elderly presenting to the emergency department after a fall is expected to increase by 47% until 2050 [1]. Moreover, 5209 of the Dutch people aged 65 and older died due to an unintentional fall in 2021 [10]. Because of its major impact on healthcare and healthcare costs, the Dutch Association of Clinical Geriatrics composed a guideline to improve fall prevention in patients living at home or in chronic care facilities [11]. The present case report describes a fatal outcome after a sustained L2 fracture due to a fall. The detrimental outcome was not expected in this initially ambulant patient with low back pain after the low-energy fall.

The high incidence of vertebral fragility fractures in elderly is mainly due to osteoporosis. In addition, ankylosing spine diseases like ankylosing spondylitis (AS) and diffuse idiopathic skeletal hyperostosis (DISH) cause the spine to be prone to highly unstable fractures [9]. In contrast to AS, DISH occurs at an older age and particularly affects the spine instead of the sacroiliac joints. Ossification of the anterior longitudinal ligaments and enthuses, as seen in this case, lead to decreased mobility until eventually complete ankylosis occurs [12, 13]. As a consequence, even minor trauma can cause fracture with high risk of instability and additional neurological deficit [14]. The prevalence of DISH in the Netherlands is approximately 17% in patients older than 50 years of age, and it is associated with obesity, type 2 diabetes, and increased life expectancy [15, 16]. The patient in this case suffered from dyslipidemia, hyperuricemia, and gout, all related to DISH [15]. A systematic review by Westerveld et al. showed that in case of DISH and AS, delay of diagnosis often occurs and awareness for the condition is highly important, as fracture instability can cause neurological deterioration after transfers or manipulation, which was also observed in the current case [14]. The Dutch guideline on acute traumatic vertebral fractures states that immobilization is necessary in any suspected thoracolumbar fracture (indicated by back pain or neurological deficit), or in case of a dangerous mechanism of injury (e.g., falls from > 3 m of height, high speed motor vehicle collisions) or known risk factors (including osteoporosis or an ankylotic spine) [17]. The patient was immobilized in a vacuum mattress after she reported back pain while walking to the gurney. Unfortunately, the diagnosis of DISH was unknown since no further tests were done after the suggestion of DISH on the radiographs in 2016, and therefore she was not immobilized immediately.

Lumbar fractures combined with severe bleeding are discussed in a number of studies. One retrospective study and three prior case reports showed accompanying lumbar artery bleeding on angiography [18–21]. In all cases, selective embolization was performed successfully. In our case, no further angiography examination was performed after initial CTA imaging, due to the agreement to discontinue further treatment. The CT-scan showed no clear image suggesting a rupture of any lumbar artery, despite the more difficult assessment due to the large hemorrhage. As shown in Fig. 1C, the bleeding extended to the epidural space causing a spinal epidural hematoma (SEH) with subsequent paralysis of the legs. Traumatic SEH occurs in between 0.5 and 1.7% of all spinal injuries, but the incidence increases to 9% in patients affected by ankylosing spondylitis or rheumatoid arthritis [22]. A retrospective case series by Tamburelli et al. investigated 7 consecutive patients with SEH. Like the patient in our case, 5 patients used oral anticoagulants and 2 were affected by DISH or AS [23]. In contrast to the patients in the case series of Tamburelli et al., the vital signs of the patient in our case deteriorated very rapidly and she died in the emergency department after discontinuing further treatment. This was most probably caused by the extension of the bleeding to the retroperitoneum and the subsequent hypovolemic shock. The use of coumarin derivates with an increased INR evidently contributed to the extensive blood loss.

In conclusion, this case report and literature review has emphasized the severity of a fall in elderly, especially in case of an ankylotic spine, as it may lead to serious neurological failure. Moreover, the use of anticoagulants contributed to the severe outcome and given its frequent use in the elderly population, one should be aware of the severe consequences. Strict spinal immobilization until proper imaging, CTA and complementary angiography should be low-threshold considerations in these patients. Apart from the co-morbidities contributing to the course of this case, morbidity and mortality are generally high in elderly who fall and this frail population is expected to increase in the future [1–3].

## Data Availability

No datasets were generated or analysed during the current study.

## Abbreviations

AS: Ankylosing spondylitis
CT: Computed tomography
CTA: Computed tomography angiography
DISH: Diffuse idiopathic skeletal hyperostosis
SEH: Spinal epidural hematoma
